# Bone Mesenchymal Stromal Cell-Derived Extracellular Vesicles Protect Articular Cartilage Through Regulating tRF-Gln-TTG-019/UBL3

**DOI:** 10.1155/mi/2705953

**Published:** 2025-06-13

**Authors:** Xialin Li, Zheng Huang, Min Shu, Guangxun Hu, Weihong Yi, Yang Duan, Songjia Ni

**Affiliations:** ^1^Department of Orthopedics, Shenzhen Nanshan People's Hospital, Shenzhen 518052, China; ^2^Department of Emergency, Shenzhen Nanshan People's Hospital, Shenzhen 518052, China; ^3^Department of Spine Surgery, Zhujiang Hospital, Southern Medical University, Guangzhou 510280, China; ^4^Department of Traumatic Orthopedics, Zhujiang Hospital, Southern Medical University, Guangzhou 510280, China

**Keywords:** articular cartilage defects, BMSCs, extracellular vesicles, tRF-Gln-TTG-019, tRNA-derived fragments, UBL3

## Abstract

The tRNA-derived fragments (tRFs) are newly discovered noncoding RNAs enriched in extracellular vesicles (EVs). However, the effects of tRFs as biomarkers have not been investigated in cartilage repair. Bone mesenchymal stromal cells (BMSCs) were isolated from male Sprague Dawley (SD) rats, and high-throughput sequencing was used to select tRFs from EVs derived from BMSC which were cultured in chondrogenic induction medium (induced) or stem cell growth medium (control). We established the rat cartilage defect model of the knee joint, in which physiological changes were examined by immunohistochemistry (IHC), to test the protective effect of BMSC-derived EVs and tRF-related molecules. Primary chondrocytes from rat knee joint treated with oxygen–glucose deprivation/reperfusion (OGD/R) were used to investigate the effect of related EVs and tRF on cell proliferation and apoptosis. BMSC-derived EVs could repair the defected cartilage of rat knee joint. OGD/R significantly reduced chondrocytes proliferation, induced chondrocyte apoptosis and inflammation, while BMSC-derived EVs reversed these effects. Additionally, tRF-Gln-TTG-019 significantly increased in EVs derived from differentiated BMSCs when compared with control group. Knockdown ubiquitin-like 3 (UBL3, a molecular target of tRF-Gln-TTG-019) inhibited chondrocytes apoptosis and inflammation induced by OGD/R, and this effect could be synergistically enhanced when co-cultured with BMSC-EVs, but the protection was partly reversed by tRF-Gln-TTG-019 inhibitor. Then we further validated that suppression of UBL3 could promote the proliferation of chondrocytes, inhibit inflammation, and enhance the repair ability of cartilage tissue in a rat cartilage defect model. Similarly to the previous results, BMSC-derived EVs could synergistically enhance these effects, while tRF-Gln-TTG-019 inhibitor weakened them. Our results indicate that tRF in BMSC-derived EVs modulates the process of cartilage repair by regulating UBL3.

## 1. Introduction

Cartilage injuries are usually caused by overload, trauma, and autoimmune diseases. In the clinic, cartilage defect is common because its limited endogenous ability to self-repair [1, 2]. At present, chondrocyte implantation and osteochondral transplantation are the main methods for cartilage defects in the clinic. However, these methods can only delay cartilage deterioration and show limited efficacy, an effective solution to restore cartilage integrity has yet to be discovered. In the past 20 years, bone marrow-derived mesenchymal stromal cells (BMSCs) have become the most widely used cell therapy for tissue regeneration [3] because of their ability to differentiate into the following cell types: osteocytes, adipocytes, and articular chondrocytes. BMSCs can repair the damaged articular cartilage by directly migrating to the injury site and differentiating into articular chondrocytes [4]; these cells exhibit residual progenitor cell regeneration in situ due to the anti-inflammatory properties [5]. In addition, BMSCs promote cell proliferation to achieve the healing of the cartilage [6, 7]. In a rat articular cartilage defect model, BMSCs inhibit the inflammation response, ameliorate the damaged cartilage microenvironment, and improve the ability to repair tissue [8]. Thus, BMSCs are quite effective in treating articular cartilage defects by inducing proliferation and differentiation of chondrocytes and inhibiting inflammation; however, the exact mechanism by which BMSCs rectify articular cartilage defects is not very clear yet.

Extracellular vesicles (EVs) are phospholipid membrane-sealing structure with a diameter of 40–160 nm [9]. In recent years, EVs derived from mesenchymal stromal cells (MSCs) were found to have therapeutic effects in the tissue repair of the heart [10], liver [11], and skin [12]. EVs derived from stem cells are a promising material for articular cartilage regeneration [13]. In 2016, for the first time, a study revealed that human embryonic MSC EVs could completely restore damaged cartilage, indicating that MSC EVs can be considered a cell-free therapeutic method for treating articular cartilage defects [14]. In the following years, several studies in animal models have reported that MSC EVs preserve chondrocyte homeostasis and relieve the pathological changes of osteoarthritis (OA) [15–18]. Moreover, human BMSCs are found to promote the regeneration of cartilage by regulating miR-23a-3p [19].

A family of small RNAs is generated by fragmenting transfer RNAs (tRNAs). These RNAs are usually 18–40 nucleotides (nt) in length and are considered as tRNA-derived fragments (tRFs) [20]. Furthermore, tRFs are classified into the following three types: precursor tRNA-derived small RNAs, mature tRFs, and halves (tRHs) [21]. Although the sequence and size of tRFs are different, they have been found to participate in many biological and physiological processes [20]. In response to a stress condition, tRFs modulate gene expression at both transcriptional and post-transcriptional levels in prokaryotes and eukaryotes [22]. Several studies have proved that the dysregulation of tRFs occurs in patients with serious diseases, such as cancer [23], Parkinson's disease [24], diabetes, and viral infections [25]. Since plasma exosomal tRF-25, tRF-38, and tRF-18 are known as biomarkers of osteoporosis, they could be used as potential predictive factors in the prognosis of osteoporosis [26]. However, previous studies have not investigated whether tRFs could be used as biomarkers of cartilage repair.

To simulate the cellular stress and injury in cartilage injuries, we performed oxygen–glucose deprivation/reperfusion (OGD/R) with BMSCs. In the present study, we report that BMSC-derived EVs protect the defects in articular cartilage of rat knee joints by regulating tRF-Gln-TTG-019, and UBL3 is the molecular target of tRF-Gln-TTG-019. Suppression of ubiquitin-like 3 (UBL3) reduces chondrocyte apoptosis, inhibits inflammation, and protects the defects in articular cartilage of rat knee joints. So BMSC-derived EVs modulate cartilage repair by regulating the expression of the tRF-Gln-TTG-019/UBL3.

## 2. Materials and Methods

### 2.1. The Isolation and Culture of BMSCs

BMSCs were isolated from 12-week-old male Sprague Dawley (SD) rats (14–21 days, 25–50 g). Briefly, bone marrow was obtained from the long bones of experimental rats. These bone marrow cells were washed with Dulbecco's Modified Eagle Medium (DMEM) containing 10% fetal bovine serum (FBS) (Gibco, Grand Island, NY, USA). Finally, these cells were centrifuged at 96 g for 3 min. The cell pellets were suspended and cultured in a medium comprising DMEM, 10% FBS, and antibiotics. Passage 3–5 cells were used for further study. All procedures of animal experiments were approved by the Committee on the Ethics of Animal Experiments of Zhujiang Hospital of Southern Medical University (LAEC-2019-006).

### 2.2. Chondrogenic Differentiation of BMSC

BMSCs (at passages 3–5) were dissociated into a single-cell suspension at a density of 2 × 10^7^ cells/mL. In total, 2 × 10^4^ cells were seeded in dishes of 6 cm diameter and allowed to form cell aggregates. A chondrogenic induction medium, as described in a previous study, was used to induce chondrogenic differentiation [27]. Briefly, cells were treated with chondrogenic induction medium, which consisted of minimum essential media (MEM). This media was supplemented with the following reagents: 10 ng/mL of recombinant human TGF-*β*1 protein (R&D Systems, Minneapolis, MN, USA), insulin transferrin selenium solution (ITS-G, Thermo Fisher Scientific, Waltham, MA, USA), 10 nM dexamethasone (Sigma–Aldrich, St. Louis, MO, USA), 50 mg/mL of ascorbic acid-2 phosphate (Sigma–Aldrich), and 40 mg/mL-proline (Sigma–Aldrich). The cells were cultured for 14 days, and the medium was replaced every 3 days. The control group cells were cultured in stem cell growth medium (DMEM, 1 mM glutamine, 100 mM sodium pyruvate, 100 mg/mL ascorbic acid).

### 2.3. EVs Isolation

EVs were isolated from control or experimental BMSCs. After 14 days of induction culture, the control or experimental BMSCs were washed by PBS three times and cultured in DMEM media containing 10% EVs-free FBS (Wuxi Puhe Biomedical Technology Co., Ltd., China) for 72 h. In total, 10 mL of the DMEM media was centrifuged at 96 g for 5 min to remove the cells. The supernatant was transferred to a new 15 mL centrifuge tube and centrifuged at 23,685 g for 30 min to remove the debris of cells. Next, incubate the supernatant with 5 mL Exo-spin buffer (EX03-8, Cell Guidance System, England) for 1 h at 4°C, and centrifuged at 23,685 g for 1 h. The EVs containing the pellets were resuspended in 200 μL of RNase-free water.

### 2.4. Transmission Electron Microscopy

Transmission electron microscopy (TEM) was performed according to a previous study [28]. Briefly, EVs were fixed with 2.5% glutaraldehyde in 0.1 M sodium carodylate solution for 1 h at 4°C. Then, they were incubated with 2% osmium tetroxide for 1 h at 4°C. After treating the EVs with graded acetone series, they were embedded in pure low-viscosity mold and baked for 24 h at 65°C. The sections of the mold were cut into 60 nm with an ultra-microtome. The slides were then double-stained with 2% uranyl acetate and lead citrate. The EVs were observed under TEM, operated at 80 kV.

### 2.5. Nanosight Tracking Analysis

The size of the EVs was determined by performing Nanosight Tracking Analysis (NTA) on a NanoSight NS300 system (Malvern Technology, Malvern, UK), which was configured with a 488 nm laser. The EVs were diluted in particle-free PBS, and they were analyzed under constant flow conditions. The data were analyzed with NTA3.1.54 software, which had a detection threshold of 5.

### 2.6. tRF Sequencing

The total RNA of EVs was extracted by Trizol reagent, and it was ligated to 3′ and 5′ small RNA adapters. The first strand of cDNA was synthesized by using random primers. The libraries were denatured, and the sequencing was performed on the Illumina NextSeq 500 system (Illumina, San Diego, Ca, USA). The abundance of tRFs was evaluated with sequencing counts. The differentially expressed tRFs were identified by the count value, determined with the R package edgeR. A publicly accessible web-based database, tRFtarget (http://trftarget.net) was used for bioinformatic analysis of the target genes affected by up- or down-regulated tRFs and presented the Gene Ontology-based biological process classification.

### 2.7. Real-Time Polymerase Chain Reaction (PCR)

The total RNA was reverse transcripted with a first-strand cDNA synthesis kit (Beyotime, Shanghai, China). The real-time PCR assays were performed with SYBR green RT-PCR kits (Applied Biosystems, Foster City, CA, USA), which were operated according to the manufacturer's protocol. The PCR reaction was carried out at 95°C for 10 min, followed by 40 cycles of denaturation (95°C for 15 s), annealing, and extension (60°C for 1 min). Table S1 enlists all primer sequences used in this study.

### 2.8. Cell Transfection

Chondrocytes were transfected with the inhibitor tRF-Gln-TTG-019, the mimic tRF-Gln-TTG-019 (Table S2 enlists all sequences), the negative control shRNA (shNC) or UBL3 shRNA (shUBL3), and the medium Lipofectermin 2000 (Invitrogen, Carlsbad, CA, USA) according to the manufacturer's instruction. The inhibitor tRF-Gln-TTG-019 (a chemically modified reverse complementary RNA), mimic tRF-Gln-TTG-019, plasmids shNC, and shUBL3 were all purchased from GenePharma (Shanghai, China). These plasmids were constructed by GenePharma in a pGPU6 vector. To perform UBL3-knockdown in vivo, the UBL3 shRNA was packaged into lentivirus (GenePharma) and injected into the knee joint of the animal. In this experiment, all transfections were repeated thrice.

### 2.9. The Isolation and Culture of Rat Chondrocytes

SD rats (6 weeks old, male) were anesthetized and sacrificed by cervical dislocation. The knee cartilage of rats was isolated and digested with 0.25% trypsin for 30 min and with 0.2% type II collagenase for 2 h. Thereafter, we prepared a culture of the tissues by mixing them with DMEM/F12 medium containing 10% FBS (Gibco). The medium was replaced after 3 days. Collagen II and toluidine blue staining were used to identify chondrocytes. Thereafter, chondrocytes were subjected to glucose deprivation and hypoxia. After 4 h, chondrocytes were exposed to glucose and a normal environment to ensure that they regained normal oxygen levels.

### 2.10. The Uptake of BMSC-Derived EVs of Chondrocytes

BMSC derived EVs were extracted and stained with PKH67 (Sigma–Aldrich). Then, the EVs (1 × 10^5^ particles/1 mL) were co-cultured with chondrocytes (1 × 10^4^), which were pretreated with a serum-free medium for 12 h. The culture was prepared at 37°C, in a 5% CO_2_ incubator and maintained for 24 hr. Then, the chondrocytes were fixed with 4% paraformaldehyde for 10 min. Subsequently, they were stained with DAPI. After washing the stained chondrocytes with PBS, we observed their images by using a fluorescent microscope (Leica). The study followed MISEV2023 recommendations [29].

### 2.11. Luciferase Assay

The potential target gene of tRF was predicted by referring to a publicly accessible web-based database, tRFtarget (http://trftarget.net) [30], miRanda, and TargetScan. The 3′ UTR of the UBL3 gene was amplified by PCR, which was performed with specific primers (Table S1) and subcloned psiCHECK-2 vector (GenePharma); the primers and the vector were subjected to double endonuclease digestion before performing PCR. The mutations in the 3′UTR of the UBL3 gene were generated by PCR and subcloned into some other vectors. The cells were co-transfected with wild-type or mutant plasmid and tRF-Gln-TTG-019 mimics. After 48 h, we collected the cells and determined their luciferase activity with the help of a commercial kit (E1500, Promega Corporation, Madison, WI, USA), which was operated according to the manufacturer's instructions.

### 2.12. Western Blot

The concentration of proteins in the cells or synovial fluid was quantified by the bicinchoninic acid assay (BCA) method. In total, 30 μg of protein was separated by performing the 10% SDS-PAGE method. The separated proteins were transferred to a polyvinylidene fluoride (PVDF) membrane, which was blocked with 5% skim milk for 1 h at room temperature. Then, the membrane containing the cells was incubated overnight at 4°C with the following antibodies: anti-NLRP3 antibody (Abcam, Cambridge, MA, USA; 1:1000 diluted), anti-pro-caspase-1 antibody (Abcam; 1:1000 diluted), anti-cleaved-caspase-1 antibody (Abcam; 1:1000 diluted), anti-MMP3 (Abcam; 1:2000 diluted), anti-MMP13 (Abcam; 1:4000 diluted) or anti-UBL3 antibody (GeneTex; 1:1000 diluted). After washing the membrane, we incubated it with HRP-linked secondary antibodies. An enhanced chemiluminescence (ECL) detection system (Millipore, Burlington, MA, USA) was used to detect the expression of proteins. The images were captured with a chemiluminescence image analysis system (Tanon 4800). The expression levels of proteins were analyzed with Image J software.

### 2.13. Ubiquitination Analysis

Cells were transfected with specific plasmid or oligo sequences. After 48 h, cells were collected and lysed in NP-40 lysis buffer and incubated with the anti-ubiquitin antibody at 4°C overnight. The next day, the cell lysate was incubated with protein G agarose beads. After washing three times with lysis buffer, the lysate was mixed with 2× loading buffer, and Western blot analysis was performed.

### 2.14. Articular Cartilage Defects in Rat Knee Joints

A total of 24 SD rats (male) were anesthetized by intraperitoneal injection of 1% pentobarbital sodium at a dose of 0.3 mL/100 g weight. Rats were randomly divided into six groups: Sham (control group, nontreatment), CI (damage group), CI+shNC (damage group + NC shRNA, 15 μL/rat with the titer of 1 × 10^8^), CI + shUBL3 (damage group + UBL3 shRNA, 15 μL/rat with the titer of 1 × 10^8^), CI+shUBL3+NC-EVs (damage group+UBL3 shRNA, 15 μL/rat with the titer of 1 × 10^8^ + EVs, 15 μL/rat with the concentration of 500 μg/mL), CI+shUBL3 + inhibitor-EVs (damage group+UBL3 shRNA, 15 μL/rat with the titer of 1 × 10^8^ + EVs, 15 μL/rat with the concentration of 500 μg/mL). For all groups that had CI, the right knee joint was routinely disinfected and made a cartilage defect by an operation [31]. The skin and fascia were cut layer by layer. The knee joint was flexed to expose the medial femoral condyle. A round full-thickness cartilage defect with a depth of 2.5 mm was drilled with an electric drill with a diameter of 1.5 mm. After modeling, the articular cavity was flushed with antibiotic injection and sutured layer by layer. Injection intervention (once per week) was performed according to above grouping at 24 h after surgery.

### 2.15. Serum Collection

The whole blood was collected from the rat orbit, and the serum was obtained by centrifuging the whole blood at 2390 g for 10 min. The supernatant was collected for further study.

### 2.16. Hematoxylin and Eosin (H&E) Staining

Eight weeks after the operation, rats were anesthetized by an intraperitoneal injection of sodium thiopental (90 mg/kg) and then sacrificed. The knee joints were disconnected from the long bones and stored in 10% formaldehyde and embedded in paraffin, then cut to 4 μm per slide. Slides were deparaffinized and rehydrated by the following steps: twice in 100% xylene for 5 min, 100% ethanol, 95% ethanol, 80% ethanol for 5 min each, and deionized H_2_O for 5 min. Slides were then stained by Hematoxylin for 3 min, Eosin for 1 min, and raised once by deionized H_2_O. Slides were dehydrated in ascending alcohol solutions (70%, 80%, 95% twice, and 100% twice) and cleared with xylene. The scoring of articular cartilage was calculated as described previously (Table 1) [32].

### 2.17. Toluidine Blue Staining

Slides were deparaffinized, rehydrated, and stained by 0.1% toluidine blue solution for 10 min at room temperature. After washing with H_2_O, slides were dehydrated in ascending alcohol solutions and cleared with xylene. The surface of the knee joint was calculated to assess the degree of bone destruction according to previous study [32]. A total severity score ranging from 0 to 12 was applied to quantify the damage of the articular cartilage in both OA and sham knees. The scoring was performed by double-blinded observers.

### 2.18. Immunohistochemistry (IHC) Assay

The rat knee joints were cut into 4-*µ*m thick sections and subsequently dewaxed and then dehydrated by immersion in a gradient alcohol series, antigen repaired, and blocked with normal goat serum for 1 h at room temperature. Ki67, PCNA, and NLRP3 antibodies (Abcam) were added to the slices and incubated at 4°C overnight. The next day, the slices were rinsed with PBS and incubated with secondary antibody for 60 min at room temperature. The slices were stained with DAB and then dehydrated and sealed with hematoxylin. The pathological features of the sections were identified under a light microscope. The staining intensity score was determined as 0 = negative, 1 = weak, 2 = moderate, and 3 = strong. The positive rate score was determined as 0 = negative, 1 = (1%–25%), 2 = (26%–50%), 3 = (51%–75%), and 4 = (76%–100%). IHC scores superior to 6 in bone and joint tissues were defined as “high expression.”

### 2.19. Statistical Analysis

All statistical data were collected and analyzed by GraphPad Prism 7.0. Statistical differences for the two groups were performed with Student's *t*-test. One-way analysis of variance was used when there was one variable, and two-way analysis of variance was used when there were two variables, followed by a post hoc Tukey test, was used to compare multiple groups. Each experiment was repeated at least three times, and the results were presented as the mean ± SD. A *p* < 0.05 was considered statistically significant.

## 3. Results

### 3.1. Characterization of BMSC and BMSC-Derived EVs

**Figure 1:**
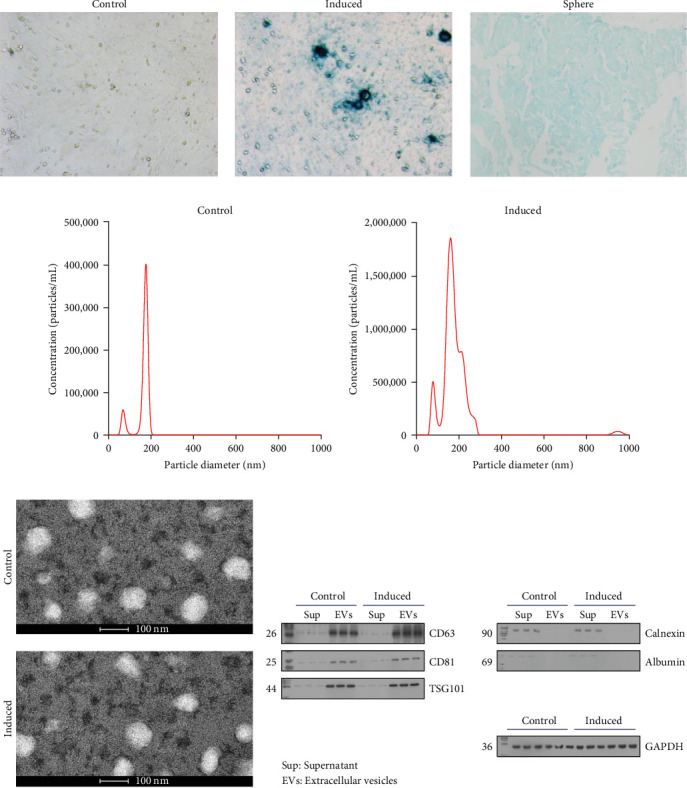
Characterization of BMSC and BMSC derived EVs. BMSCs were cultured in a chondrogenic induction medium and stained by Alcian blue (A). The images were shown (200×). The EVs were extracted from control or induced BSMCs. The characteristics of EVs were identified by nanosight (B) and TEM (C). The expression changes of CD63, CD81, TSG101, Calnexin, and albumin were certified using western blot assay (D), *n* = 3. BMSC, bone mesenchymal stromal cell; EVs, extracellular vesicles; TEM, transmission electron microscopy.

BMSCs were isolated and analyzed by flow cytometry for cell markers. As shown in Figure S1, the proportion of CD29-positive cells and CD44-positive cells is 97.9% and 95.5%, while CD34-positive cells only account for 1.2%, and CD45-positive cells account for 0.9%. Alcian blue staining indicated the differentiation of BMSCs to mature chondrocytes (Figure 1A). The size of EVs extracted from control and induced BSMCs was determined by Nanosight and TEM (Figure 1B,C). The EVs were around 100–200 nm spherical particles with a complete membrane structure. We further confirmed the EVs through Western blot analysis of EV-specific markers CD63, CD81, and TSG101 and negative indicators Calnexin and albumin (Figure 1D).

### 3.2. BMSC-Derived EVs Ameliorate the Defects of Articular Cartilage in Rat Knee Joints

**Figure 2:**
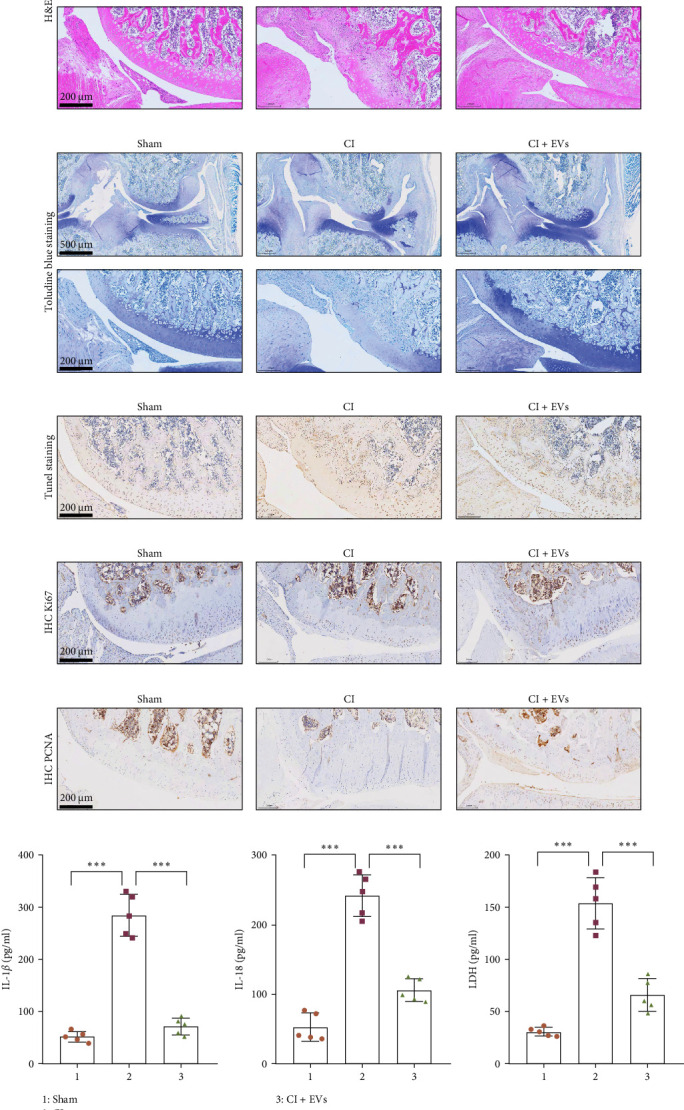
BMSC-derived EVs protect the defects in articular cartilage of rat knee joints. A model of defects in articular cartilage was established by a round full-thickness cartilage defects with a depth of 2.5 mm. The knee joint cartilage was collected and stained by H&E (A), toluidine blue (B), and TUNEL (C). The arrow indicates the location of the defect. The expression levels of Ki67 and PCNA were detected by immunohistochemistry assay (D, E). The synovial fluid was separated and the content of IL-1b, IL-18, and LDH were detected by ELISA (F). *⁣*^*∗∗∗*^*p* < 0.001, *n* = 5. BMSC, bone mesenchymal stromal cell; EVs, extracellular vesicles; H&E, hematoxylin and eosin.

A model to mimic the articular cartilage injury (CI) was established in the right knee joint of rats, and BMSC-derived EVs were injected into the cartilage defect of the knee joint. Articular cartilage was significantly thinner in the CI group than in the control group, as verified by H&E and toluidine blue staining. We found that the articular cartilage surface becomes smooth and the arrangement of chondrocytes becomes tighter in BMSC-derived EVs injections group (Figure 2A,B). In addition, TUNEL staining showed that the TUNEL positive cells of the articular cartilage were increased in CI rats (Figure 2C). Immunohistochemical results showed that the number of Ki67^+^ cells and PCNA^+^ cells in articular cartilage was increased in the CI+EVs group when compared with that in the CI group (Figure 2C,D). At the same time, the expression of inflammatory factors in synovial fluid, including IL-1*β*, IL-18, and LDH, increased significantly in the CI group but decreased when BMSC-derived EVs were locally injected (Figure 2F).

### 3.3. Differentially Expressed tRFs in BMSC Derived EVs

To find out the mechanism of the protected function of BMSC-derived EVs, a high-throughput sequencing technique was used to determine the differential expression of tRFs in EVs released by both pre-differentiated and post-differentiated BMSCs. The scatter and volcano plots indicate the change in expression levels of tRFs (Figure S2A,B). A total of 65 tRFs had significantly changed (22 tRFs were up-regulated, and 43 tRFs were down-regulated, fold change >1.5 and *p* < 0.05, the *p*-value was corrected by Benjamini–Hochberg, and the FDR value was less than 0.05) in EVs released by post-differentiated BMSCs compared with the control cells (Table S3). The differential expression of tRFs in EVs released by control or post-differentiated BMSCs are also represented in a heatmap (Figure S2A). We utilized a publicly accessible web-based database, tRFtarget (http://trftarget.net), for bioinformatic analysis of the target genes affected by up- or down-regulated tRFs and presented the Gene Ontology-based biological process classification (Figure S2B). The signal transduction pathway of the differentiated gene revealed that the MAPK signaling pathway and expression of cytokine receptors were significantly changed when tRF levels declined (Figure S3A,B).

### 3.4. EVs Derived From BMSC Protect OGD/R Caused Apoptosis and Inflammation of Chondrocytes Through Transferring tRF-Gln-TTG-019

**Figure 3:**
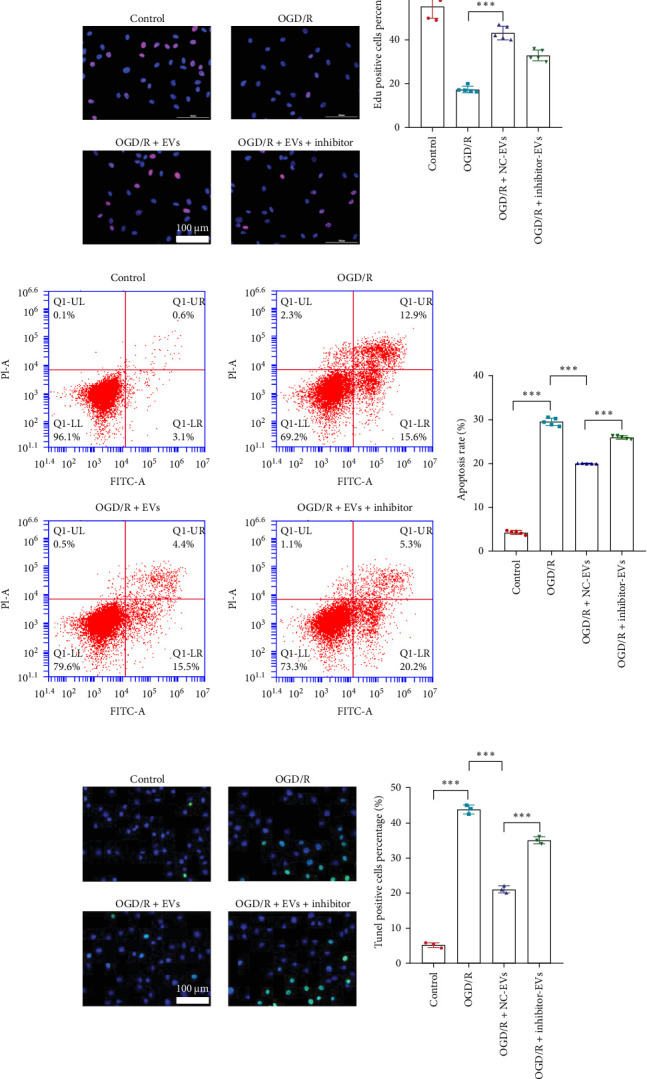
tRF-Gln-TTG-019 is regulated by BMSC-derived EVs to protect chondrocytes apoptosis and inflammation. (A) RNA was extracted from EVs derived from control or post-differentiated BMSCs. The differentially expressed tRFs were determined by high throughput sequencing. (B) The differentially expressed tRFs were validated by real-time PCR. Primary rat chondrocytes were treated by OGD/R and co-cultured with EVs derived from BMSCs and transfected with NC (OGD/R+EVs) or tRF-Gln-TTG-019 inhibitor (OGD/R+EVs+inhibitor), *n* = 5. After 48 h, cells were collected for PKH26 staining (C). The expression of tRF-Gln-TTG-019 was determined by real-time PCR, *n* = 5 (D). The cell proliferation was tested by CCK8 (E) and EdU staining (F). The cell apoptosis was determined by Annexin V/PI double staining (G) and TUNEL (H) assays. The content of IL-1b, IL-18, and LDH were detected by ELISA (I). The expression of NLRP3 and Caspase-1 was examined by Western blot (J). (K) Total cell lysates were immunoprecipitated with anti-UBL3 antibody and subsequently immunoblotted with anti-ubiquitin antibody. The expression of NLRP3 was detected by Western blot. *⁣*^*∗*^*p* < 0.05, *⁣*^*∗∗*^*p* < 0.01, *⁣*^*∗∗∗*^*p* < 0.001. BMSC, bone mesenchymal stromal cell; EVs, extracellular vesicles; tRF, tRNA-derived fragment.

As shown in Figure 3A,B, the expression of tRF-Gln-TTG-019 was significantly increased in EVs derived from post-differentiated BMSCs when compared to other tRFs. To examine the function of tRF-Gln-TTG-019 in chondrocytes, the primary rat chondrocytes were isolated from knee cartilage, which were characterized through the presence of type II collagen using immunofluorescence and Toluidine blue staining methods (Figure S4A,B). The inhibitor of tRF-Gln-TTG-019 was designed to regulate the expression of tRF-Gln-TTG-019, and the inhibition efficiency was confirmed by real-time PCR, whereby the expression of tRF-Gln-TTG-019 was found to be significantly reduced (Figure S4C). Moreover, chondrocytes were co-cultured with EVs derived from post-differentiated BMSCs following OGD/R treatment. The internalization of EVs into chondrocytes was confirmed by RKH26 staining (Figure 3C). In addition, the expression of tRF-Gln-TTG-019 was significantly reduced with OGD/R treatment but restored by co-culturing with EVs (Figure 3D). Transfection of tRF-Gln-TTG-019 inhibitor into the chondrocytes reduced the expression of tRF-Gln-TTG-019 compared to ODG/R+BMSC EV-treated cells (Figure 3D). The reduction in cell proliferation caused by OGD/R treatment was reversed by co-culturing the chondrocytes with EVs (Figure 3E,F). Additionally, the tRF-Gln-TTG-019 inhibitor reduced the protective role of EVs (Figure 3E,F). Cell apoptosis in OGD/R-treated chondrocytes was also significantly reduced with the introduction of EVs (Figure 3G,H), although the addition of tRF-Gln-TTG-019 inhibitor ceased the anti-apoptotic effects provided by the EVs (Figure 3G,H). Furthermore, the OGD/R-caused inflammation was also observed by increasing the expression of IL-1*β*, IL-18, and LDH (Figure 3I). It is reported that Nod-like receptor protein 3 (NLRP3) inflammasomes are activated in response to cellular stress conferred by various infections and environmental irritants [33]. Our results showed that the expression of NLRP3 and Caspase-1 was increased in chondrocytes under OGD/R treatment but minimized when co-cultured with the extracted EVs (Figure 3J and Figure S5A). However, the tRF-Gln-TTG-019 inhibitor abrogated the effects of EVs. Also, we detected the ubiquitination of NLRP3 under OGD/R station. As shown in Figure 3K, the expression of NLRP3 was increased by decreasing the ubiquitination level of NLRP3 under OGD/R treatment, while EVs derived from BMSCs induced the ubiquitination of NLRP3. The inhibitor of tRF-Gln-TTG-019 partly reversed this effect. In addition, it was found that UBL3, an ubiquitination regulatory factor, may have a binding relationship with NLRP3 protein.

### 3.5. tRF-Gln-TTG-019 Protects Chondrocytes Apoptosis and Inflammation by Targeting UBL3

**Figure 4:**
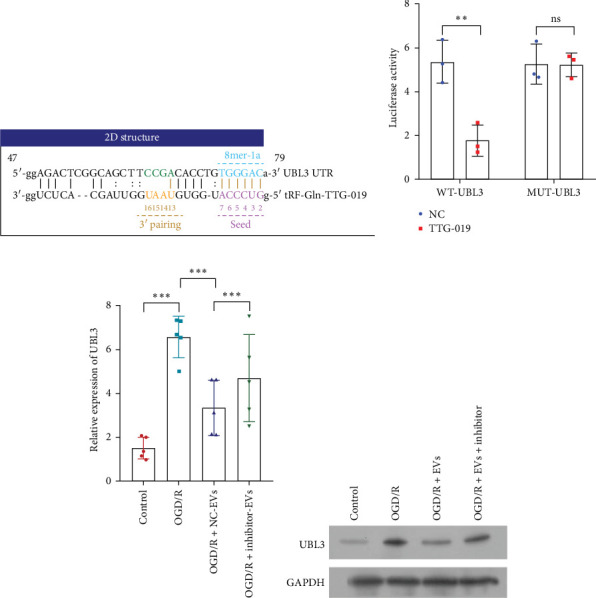
tRF-Gln-TTG-019 protects chondrocytes apoptosis and inflammation by targeting UBL3. (A) The predicated binding sites between tRF-Gln-TTG-019 and UBL3 untranslated region were shown. (B) Chondrocytes were co-transfected with UBL3 (wild type) and tRF-Gln-TTG-019 or mutant form of UBL3 and tRF-Gln-TTG-019. Cells were collected after transfection of 48 h. The luciferase activity was shown. (C, D) The mRNA and protein expression of UBL3 was determined by real-time PCR and Western blot. *⁣*^*∗∗*^*p* < 0.01, *⁣*^*∗∗∗*^*p* < 0.001. ns, no significant difference; tRF, tRNA-derived fragment.

**Figure 5:**
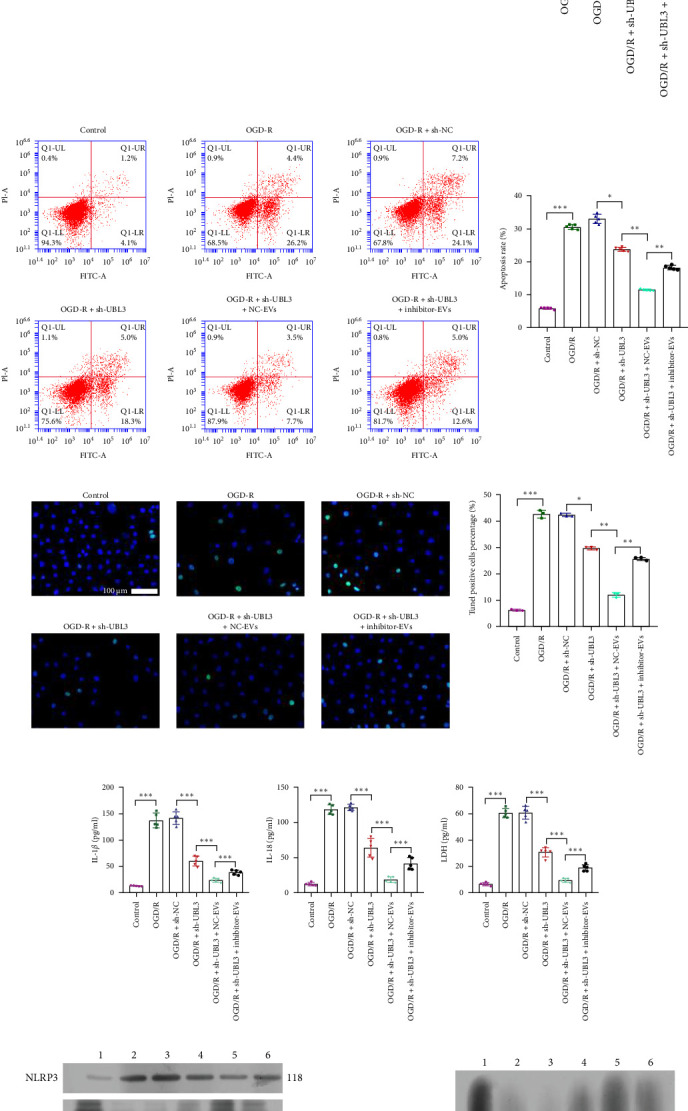
Knockdown UBL3 suppresses the modulated function of tRF-Gln-TTG-019. Primary rat chondrocytes were treated with OGD/R, 4 h later, cells were transfected with NC or shUBL3 or co-transfected with shUBL3 and NC-EVs or inhibitor of tRF-Gln-TTG-019. The expression of UBL3 was determined by real-time PCR, *n* = 5 (A). The cell proliferation was tested by CCK8 (B) and EdU staining (C). The cell apoptosis was determined by Annexin V (D) and TUNEL (E) assays. The content of IL-1b, IL-18, and LDH were detected by ELISA (F). The expression of NLRP3, Caspase-1, MMP3, and MMP13 was examined by Western blot (G). (H) His-NLRP3 was overexpressed in chondrocytes. Total cell lysates were immunoprecipitated with anti-UBL3 antibody and subsequently immunoblotted with anti-ubiquitin antibody. The expression of His-NLRP3 was detected by Western blot. (I) Sulfated glycosaminoglycan (sGAGs) released from the culture medium were detected by ELISA. *⁣*^*∗*^*p* < 0.05, *⁣*^*∗∗*^*p* < 0.01, *⁣*^*∗∗∗*^*p* < 0.001. tRF, tRNA-derived fragment.

To further investigate the targeting downstream gene of tRF-Gln-TTG-019, bioinformatic studies were conducted to show that the untranslated region of UBL3 has a potential binding site with tRF-Gln-TTG-019 (Figure 4A). A luciferase reporter assay was performed to validate this interaction, which revealed that tRF-Gln-TTG-019 significantly affected UBL3 luciferase activity (Figure 4B). Consistently, UBL3 expression was induced by OGD/R treatment in chondrocytes but mitigated by BMSC derived EVs. The addition of tRF-Gln-TTG-019 inhibitor in OGD/R-treated cells with extracted EVs showed up-regulation of UBL3 (Figure 4C,D). As a binding target of tRF-Gln-TTG-019, we determined the involvement of UBL3 in OGD/R-induced cell apoptosis and inflammation. Chondrocytes were first transfected with NC or UBL3 shRNA, before being treated with OGD/R and EVs. The expression of UBL3 was determined by real-time PCR. UBL3 was significantly enhanced by OGD/R, although inhibition of UBL3 by shRNA reduced it under OGD/R conditions. In addition, co-treatment of BMSC-derived EVs and UBL3 shRNA synergistically inhibited the expression of UBL3, while tRF-Gln-TTG-019 inhibitor reversed this effect (Figure 5A). Under OGD/R condition, both shUBL3 and BMSC-EVs could promote cell proliferation, and the proliferation rate of BMSC-EVs was higher, but the tRF-Gln-TTG-019 inhibitor could partially inhibit the proliferation of BMSC-EVs (Figure 5B,C). OGD/R induced cell apoptosis was alleviated by UBL3 inhibition and BMSC-EVs, but the protect effect of BMSC-EVs was weakened by the inhibitor of tRF-Gln-TTG-019 (Figure 5D,E). Moreover, the expression of inflammatory factors, such as IL-1, IL-18, and LDH induced by OGD/R, was inhibited by UBL3 shRNA (Figure 5F). More importantly, BMSC derived EVs further inhibited the expression of inflammatory factors, but this effect was partly reversed by tRF-Gln-TTG-019 inhibitor (Figure 5F). We also detected the expression of NLRP3 and Caspase-1 and found that OGD/R induced NLRP3, Caspase-1, MMP3,and MMP13 expression were reduced by UBL3 shRNA or BMSC-derived EVs, while tRF-Gln-TTG-019 inhibitor could alleviate these effects (Figure 5G and Figure S5B). Furthermore, the ubiquitination of NLRP3 was inhibited by OGD/R, but enhanced by UBL3 shRNA or BMSC-derived EVs under OGD/R treatment. The inhibitor of tRF-Gln-TTG-019 abolished the effects caused by UBL3 shRNA or EVs (Figure 5H). In addition, the sulfated GAGs released in the culture medium were increased in the OGD/R group, while knocking down UBL3 and incubation with EVs partially weakened this effect (Figure 5I). The results suggest that UBL3 expression may be regulated by tRF-Gln-TTG-019, which may mediate protective effects on chondrocytes under OGD/R treatment.

### 3.6. Knockdown UBL3 Ameliorate the Defects of Articular Cartilage in Rat Knee Joints

**Figure 6:**
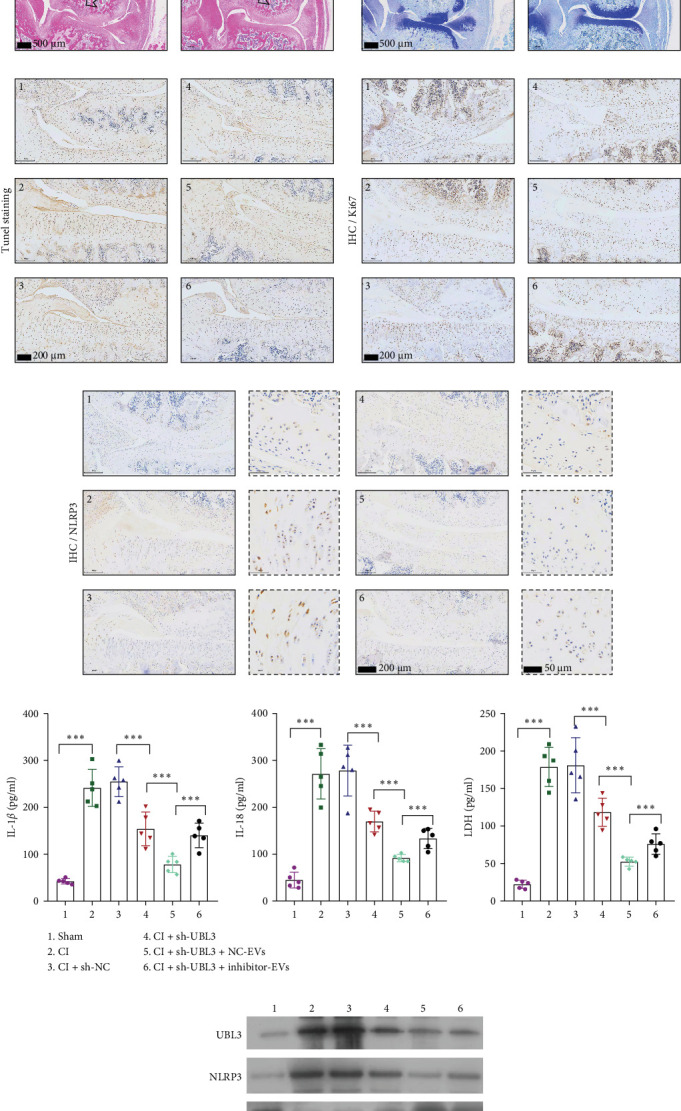
UBL3 shRNA or BMSC-derived EVs ameliorate the defects of articular cartilage in rat knee joints. A model of defects in articular cartilage was established by a round full-thickness cartilage defects with a depth of 2.5 mm. The knee joint cartilage was collected and stained by H&E (A), toluidine blue (B), and TUNEL (C). Immunohistochemistry was used to detect Ki67 (D) and NLRP3 (E) (200× magnification). The synovial fluid was separated and subjected to ELISA assay (F). The protein levels of UBL3, NLRP3, and Caspase-1 in synovial fluid were tested by Western blot (G). (1) Sham, (2) CI, (3) CI+shNC, (4) CI+shUBL3, (5) CI+shUBL3+NC-EVs, (6) CI+shUBL3+inhibitor-EVs. *⁣*^*∗∗∗*^*p* < 0.001, *n* = 5. BMSC, bone mesenchymal stromal cell; EVs, extracellular vesicles; H&E, hematoxylin and eosin.

To further validate whether shUBL3 and tRF-Gln-TTG-019 injections could reduce the injury of articular cartilage, a model to mimic the defects of articular cartilage was established in the right knee joint of rats. Articular cartilage was verified by H&E, toluidine blue staining, TUNEL staining, and IHC staining of Ki67 and NLRP3. Reduced chondrocyte proliferation was observed in CI group, but it could be partially reversed by the injection containing UBL3 shRNA or BMSC-derived EVs. However, the inhibitor of tRF-Gln-TTG-019 reduced the protective effect provided by UBL3 shRNA (Figure 6A–E and Figure S5D–H). Moreover, the level of inflammatory factors IL-1*β*, IL-18, and LDH in synovial fluid indicated that articular cartilage defects induced inflammation was repressed by UBL3 shRNA and BMSC derived EVs, and this effect could be reversed by the inhibitor of tRF-Gln-TTG-019 (Figure 6F). The expression level of NLRP3 in synovial fluid was determined by Western blot. The results revealed that NLRP3 expression increased in articular cartilage defects, and suppression of UBL3 through BMSC-derived EVs partly decreased the expression of NLRP3 (Figure 6G and Figure S5C). These results indicated that inhibition of UBL3 may play protective roles in articular cartilage defects in rat knee joints.

## 4. Discussion

**Figure 7:**
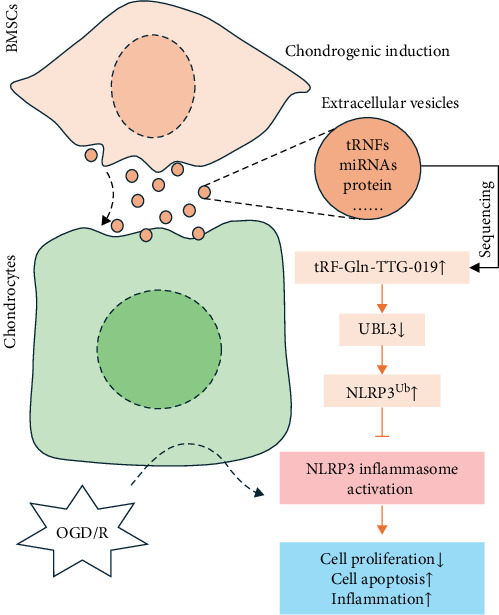
Schematic diagram of the mechanism of bone marrow mesenchymal stromal cell-derived extracellular vesicles protecting articular cartilage by delivering tRF-Gln-TTG-019. tRF, tRNA-derived fragment.

Adult articular cartilage has limited capacity for regeneration after injury [34]. Cell therapy, such as stem cell therapy, is becoming the potential therapeutic method for cartilage repair [35]. Recent evidences from both adult MSCs and human pluripotent stem cells (hPSCs) have supported the promising results for cartilage repair in clinical studies [36–38]. As these stem cells possess self-renewal capacity and the ability to differentiate into various cell types, transplantation of BMSCs provides a promising treatment option to repair the damaged articular cartilage [4]. Actually, paracrine secretion of trophic factors, such as EVs, attributed to the efficacy of several types of stem cell-based tissue patches for articular cartilage regeneration [13]. EVs derived from human amniotic fluid cells (AFSC) have shown a strong ability to repair cartilage defects in an OA animal model induced by monosodium Iodoacetate (MIA) [39]. Human MSC EVs combined with hyaluronic acid (HA) were observed to have enhanced functional cartilage repair ability [40]. Here, we found that BMSC-derived EVs can prevent articular cartilage defects in an in vitro OGD/R model and an in vivo rat model, consistent with the previous studies. Nucleotide sequencing revealed that tRF-Gln-TTG-019 was highly expressed in post-differentiated BMSC-EVs. Through an in vitro OGD/R model, we show that tRF-Gln-TTG-019 presented in BMSC-EVs mediates a protective effect on OGD/R-treated chondrocytes (Figure 7). However, it is possible that increased expression of tRF-Gln-TTG-019 was induced by TGF*β*1 or other components instead of chondrogenic differentiation itself.

As potential biomarkers for diagnosis and prognosis of various diseases [41]. EVs are enriched in proteins, noncoding RNAs such as microRNAs and tRFs [42]. Several studies have shown that the stem cell-derived EVs protect articular cartilage defects by noncoding RNAs. Umbilical cord mesenchymal stem cells-derived small EVs (hUC-MSCs-sEVs) promote the migration, proliferation, and differentiation of chondrocytes, and human BMSCs induce cartilage regeneration by regulating miR-23a-3p [19]. Intrapatellar fat pad (IPFP) MSCs-derived EVs prevent articular cartilage from damage and repair gait abnormalities in osteoarthritic (OA) mice through miR-100-5p-regulated inhibition of the mTOR-autophagy pathway [18]. In this study, we have shown that another type of noncoding RNAs, tRF-Gln-TTG-019 present in EVs derived from BMSC, is involved in providing the protective effect on articular cartilage. A recent study showed that oxygen–glucose deprivation of chondrocytes caused cell apoptosis and reduced proliferation [43]. Similarly, we found OGD/R accelerated chondrocytes apoptosis significantly in vitro and BMSC-EVs containing tRF-Gln-TTG-019 alleviated this process. The MAPK signaling pathway plays important roles in apoptosis, inflammation [44], differentiation, survival [45], and extracellular matrix (ECM) production of chondrocytes [46], suggesting that these tRFs may be involved in the proliferation, differentiation, ECM production, and chondrocyte-induced inflammation. In vitro experimental results indicated that the EVs derived from BMSC prevent cell apoptosis and inflammation caused by OGD/R treatment by regulating tRF-Gln-TTG-019 expression.

Hypoxia is a common phenomenon during bone development and repair, especially during the stages of cartilage formation and differentiation. Zhao et al. [47] investigated the therapeutic efficacy of hypoxia-treated adipose mesenchymal stem cell-derived exosomes on lumbar facet joint OA, revealing their enhanced protective effects in alleviating articular cartilage degradation, subchondral bone remodeling abnormalities, and spinal pain. However, how chondrocytes survive in hypoxia deserves further study. Multiple studies have revealed the important role of hypoxic environment in the differentiation process of chondrocytes [48, 49]. Li et al. [50] found that hypoxia promotes maintenance of the chondrogenic phenotype in rat growth plate chondrocytes through the HIF-1*α*/YAP signaling pathway. However, the specific mechanism of hypoxia on chondrogenic differentiation still needs further exploration. In the presented study, we found that hypoxia altered the composition of EVs in BMSC. In fact, the source of EVs is multifaceted. M2 macrophage-derived exosome and dendritic cell-derived exosome both can regulate cell differentiation [51, 52]. How hypoxia affects the expression level of exosomal tRF-Gln-TTG-019 may be our next research plan.

As the target for tRF-Gln-TTG-019, UBL3 is a ubiquitin-like protein, which was first identified in *Drosophila*. It acts as a post-translational modification factor to regulate protein sorting to EVs [53]. Recently, UBL3 was reported to be upregulated in human prostate cancer cells and act as a predictor for relapse and survival in patients with cervical carcinoma or esophageal cancer [54, 55]. In addition, a new study found that UBL3 functions importantly in immune responses through intervening the ubiquitination of MARCH1 substrates [56]. In this study, inhibition of UBL3 in chondrocytes prevented cell apoptosis caused by OGD/R and reduced inflammation in articular cartilage defects in rat model. Our data suggested that tRF-Gln-TTG-019 and its target UBL3 played protective roles in articular CI.

To OA, inflammatory mediators contribute to rupture of cartilage homeostasis, which ultimately results in cartilage damage [57]. Inflammasome signaling molecules, such as NLRP3, are involved in the progression of OA [58]. As an inflammasome factor, NLRP3 activates Caspase-1 and leads to the secretion of inflammatory cytokines IL-1*β*, IL-18, and tumor necrosis factor-alpha [59, 60]. Additionally, NLRP3 and the IL-1*β* activator caspase-1 are expressed in damaged cartilage and in the synovial tissue in OA patients [61–63]. In this study, BMSC-EVs or knockdown UBL3 could inhibit the inflammation caused by OGD/R or articular cartilage defects. These data suggested that BMSC-EVs or tRF-Gln-TTG-019 could protect articular cartilage probably through suppressing inflammation. However, one of the limitations of our study was the absence of using human primary chondrocytes or human cartilage explants. In vitro experiments, there were also differences between OGD/R model and CI model recognized by most scholars. The CI model induced by stimulating cells with IL-1*β* or TNF-*α* may make the results more convincing.

In conclusion, our study reported that tRF-Gln-TTG-019 is involved in BMSC-EVs and can modulate cartilage repair by regulating UBL3. BMSC-EVs and knockdown UBL3 could protect chondrocytes from apoptosis and inflammation synergistically. Inhibition of UBL3 promoted repairing defects in articular cartilage of rat knee joints.

## Figures and Tables

**Table 1:** Standard of Wakitani.

Index	Tissue expression	Scores
Cell morphology	Hyaline cartilage Hyaline cartilage is the main content Fibrocartilage is the main content Contains a small amount of cartilage No cartilage	0 1 2 3 4

Matrix staining	Normal Stain light Stain decrease significantly No stain	0 1 2 3

Cartilage surface^a^	Smooth (>3/4) Coarser (1/2–3/4) Irregular (1/4–1/2) Especially rough (<1/4)	0 1 2 3

Cartilage thickness^b^	>2/3/0 1/3–2/3/1 <1/3	0 1 2

The degree of binding between the filling and the surrounding cartilage	>2/3 1/3–2/3 <1/3	0 1 2

Total scores	—	14

^a^The ratio of smooth surface to total defect area of cartilage.

^b^The ratio of the average thickness of the packed cartilage to the thickness of the surrounding cartilage.

## Data Availability

The data that support the findings of this study are available from the corresponding author upon reasonable request.

## Nomenclature

BMSCs: Bone marrow mesenchymal stromal cells
tRFs: tRNA-derived fragments
OGD/R: Oxygen–glucose deprivation/reperfusion
UBL3: Ubiquitin-like 3
MSCs: Mesenchymal stem cells
OA: Osteoarthritis
TEM: Transmission electron microscopy
H&E: Hematoxylin and eosin
PI: Propidium iodide
NLRP3: Nod-like receptor protein 3
hPSCs: Human pluripotent stem cells
AFSC: Amniotic fluid cells
HA: Hyaluronic acid
MIA: Monosodium iodoacetate
IPFP: Intrapatellar fat pad.

## Supporting Information

Supporting Information 1 Table S1. Primer sequences.

Supporting Information 2 Table S2. Sequences of inhibitor, mimics and shRNA.

Supporting Information 3 Table S3. Differentially_Expressed_tRF.

Supporting Information 4 Figure S1. Identification of rat BMSCs. Rat BMSCs were isolated and performed flow cytometry assay (FITC labeled CD29, CD34, CD44, CD45, and CD90). Negative control (NC) was incubated with PBS.

Supporting Information 5 Figure S2. Heat map and GO TERM assay. (A) Differentially expressed tRFs were displayed by heat map. The color in the panel represented the relative expression level (log2-transformed). The color scale is shown below: blue represented an expression level below the mean, and red represented an expression level above the mean. The colored bar top at the top panel showed the sample group, and the colored bar at the right side of the panel indicated the divisions which were performed using K-means. (B) GO TERM analysis in up (upper panel) or down-regulated (lower panel) tRFs.

Supporting Information 6 Figure S3. Signaling pathway assay. (A and B) Signal pathway analysis of target genes in up (left panel) or down-regulated (right panel) tRFs.

Supporting Information 7 Figure S4. Suppression of tRF-Gln-TTG-019 expression by the specific inhibitor. (A and B) Primary rat chondrocytes were isolated from knee cartilage and identified by Toluidine blue staining and type collagen II by immunofluorescence. (C) Chondrocytes were transfected with NC shRNA or Dicer shRNA. The expression of tRF-Gln-TTG-019 was detected by real-time PCR (left panel). Rat chondrocytes were transfected with NC or tRF-Gln-TTG-019 inhibitor, the expression of tRF-Gln-TTG-019 was detected by real-time PCR (right panel). *⁣*^*∗∗∗*^*p* < 0.001.

Supporting Information 8 Figure S5. Statistical analysis of Western blot and IHC. (A) The protein expressions were quantified from three independent experiments. The statistical results were shown. A was related to Figure 3J,K; B was related to Figure 5G,H; C was related to Figure 6G. (D and E) The scoring of H&E and Toluidine blue in Figure 6A,B. (F and H) Quantified data of TUNEL staining and IHC results in Figure 6C–E. *⁣*^*∗*^*p* < 0.05; *⁣*^*∗∗*^*p* < 0.01; *⁣*^*∗∗∗*^*p* < 0.001.

Supporting Information 9 Detailed description of some experimental methods.

## References

[1] Bhosale A. M., Richardson J. B. (2008). Articular Cartilage: Structure, Injuries and Review of Management. *British Medical Bulletin*.

[2] Le H., Xu W., Zhuang X., Chang F., Wang Y., Ding J. (2020). Mesenchymal Stem Cells for Cartilage Regeneration. *Journal of Tissue Engineering*.

[3] Richardson S. M., Kalamegam G., Pushparaj P. N. (2016). Mesenchymal Stem Cells in Regenerative Medicine: Focus on Articular Cartilage and Intervertebral Disc Regeneration. *Methods*.

[4] Bornes T. D., Adesida A. B., Jomha N. M. (2014). Mesenchymal Stem Cells in the Treatment of Traumatic Articular Cartilage Defects: A Comprehensive Review. *Arthritis Research & Therapy*.

[5] Gupta P. K., Das A. K., Chullikana A., Majumdar A. S. (2012). Mesenchymal Stem Cells for Cartilage Repair in Osteoarthritis. *Stem Cell Research & Therapy*.

[6] He L., He T., Xing J. (2020). Bone Marrow Mesenchymal Stem Cell-Derived Exosomes Protect Cartilage Damage and Relieve Knee Osteoarthritis Pain in a Rat Model of Osteoarthritis. *Stem Cell Research & Therapy*.

[7] Lam A. T. L., Reuveny S., Oh S. K.-W. (2020). Human Mesenchymal Stem Cell Therapy for Cartilage Repair: Review on Isolation, Expansion, and Constructs. *Stem Cell Research*.

[8] Liu Y., Li M., Yin Z., Zhou S., Qiu Y. (2020). SUMO-Modified Bone Marrow Mesenchymal Stem Cells Promoted the Repair of Articular Cartilage in Rats. *Cell Biology International*.

[9] Choi D. S., Kim D. K., Kim Y. K., Gho Y. S. (2013). Proteomics, Transcriptomics and Lipidomics of Exosomes and Ectosomes. *Proteomics*.

[10] Lai R. C., Arslan F., Lee M. M. (2010). Exosome Secreted by MSC Reduces Myocardial Ischemia/Reperfusion Injury. *Stem Cell Research*.

[11] Tan C. Y., Lai R. C., Wong W., Dan Y. Y., Lim S.-K., Ho H. K. (2014). Mesenchymal Stem Cell-Derived Exosomes Promote Hepatic Regeneration in Drug-Induced Liver Injury Models. *Stem Cell Research & Therapy*.

[12] Zhang B., Yin Y., Lai R. C., Tan S. S., Choo A. B. H., Lim S. K. (2014). Mesenchymal Stem Cells Secrete Immunologically Active Exosomes. *Stem Cells and Development*.

[13] Liu X., Yang Y., Li Y. (2017). Integration of Stem Cell-Derived Exosomes With in Situ Hydrogel Glue as a Promising Tissue Patch for Articular Cartilage Regeneration. *Nanoscale*.

[14] Zhang S., Chu W. C., Lai R. C., Lim S. K., Hui J. H. P., Toh W. S. (2016). Exosomes Derived From Human Embryonic Mesenchymal Stem Cells Promote Osteochondral Regeneration. *Osteoarthritis and Cartilage*.

[15] Cosenza S., Ruiz M., Toupet K., Jorgensen C., Noël D. (2017). Mesenchymal Stem Cells Derived Exosomes and Microparticles Protect Cartilage and Bone From Degradation in Osteoarthritis. *Scientific Reports*.

[16] Wang Y., Yu D., Liu Z. (2017). Exosomes From Embryonic Mesenchymal Stem Cells Alleviate Osteoarthritis Through Balancing Synthesis and Degradation of Cartilage Extracellular Matrix. *Stem Cell Research & Therapy*.

[17] Mao G., Zhang Z., Hu S. (2018). Exosomes Derived From miR-92a-3p-Overexpressing Human Mesenchymal Stem Cells Enhance Chondrogenesis and Suppress Cartilage Degradation via Targeting WNT5A. *Stem Cell Research & Therapy*.

[18] Wu J., Kuang L., Chen C. (2019). MiR-100-5p-Abundant Exosomes Derived From Infrapatellar Fat Pad MSCs Protect Articular Cartilage and Ameliorate Gait Abnormalities via Inhibition of mTOR in Osteoarthritis. *Biomaterials*.

[19] Hu H., Dong L., Bu Z. (2020). MiR-23a-3p-Abundant Small Extracellular Vesicles Released From Gelma/Nanoclay Hydrogel for Cartilage Regeneration. *Journal of Extracellular Vesicles*.

[20] Tosar J. P., Cayota A. (2020). Extracellular tRNAs and tRNA-Derived Fragments. *RNA Biology*.

[21] Li S., Xu Z., Sheng J. (2018). tRNA-Derived Small RNA: A Novel Regulatory Small Non-Coding RNA. *Genes*.

[22] Krishna S., Raghavan S., DasGupta R., Palakodeti D. (2021). TRNA-Derived Fragments (tRFs): Establishing Their Turf in Post-Transcriptional Gene Regulation. *Cellular and Molecular Life Sciences*.

[23] Zhu L., Liu X., Pu W., Peng Y. (2018). tRNA-Derived Small Non-Coding RNAs in Human Disease. *Cancer Letters*.

[24] Prehn J. H. M., Jirström E. (2020). Angiogenin and tRNA Fragments in Parkinson’s Disease and Neurodegeneration. *Acta Pharmacologica Sinica*.

[25] Jiang P., Yan F. (2019). TiRNAs & tRFs Biogenesis and Regulation of Diseases: A Review. *Current Medicinal Chemistry*.

[26] Zhang Y., Cai F., Liu J. (2018). Transfer RNA-Derived Fragments as Potential Exosome tRNA-Derived Fragment Biomarkers for Osteoporosis. *International Journal of Rheumatic Diseases*.

[27] Lu T.-J., Chiu F.-Y., Chiu H.-Y., Chang M.-C., Hung S.-C. (2017). Chondrogenic Differentiation of Mesenchymal Stem Cells in Three-Dimensional Chitosan Film Culture. *Cell Transplantation*.

[28] Jung M. K., Mun J. Y. (2018). Sample Preparation and Imaging of Exosomes by Transmission Electron Microscopy. *Journal of Visualized Experiments*.

[29] Welsh J. A., Goberdhan D. C. I., O’Driscoll L. (2024). Journal of Extracellular Vesicles. *Minimal Information for Studies of Extracellular Vesicles (MISEV2023): From Basic to Advanced Approaches*.

[30] Li N., Shan N., Lu L., Wang Z. (2021). TRFtarget: A Database for Transfer RNA-Derived Fragment Targets. *Nucleic Acids Research*.

[31] Lee H. H., O’Malley M. J., Friel N. A., Chu C. R. (2013). Effects of Doxycycline on Mesenchymal Stem Cell Chondrogenesis and Cartilage Repair. *Osteoarthritis and Cartilage*.

[32] Pinamont W. J., Yoshioka N. K., Young G. M. (2020). Standardized Histomorphometric Evaluation of Osteoarthritis in a Surgical Mouse Model. *Journal of Visualized Experiments*.

[33] Swanson K. V., Deng M., Ting J. P.-Y. (2019). The NLRP3 Inflammasome: Molecular Activation and Regulation to Therapeutics. *Nature Reviews Immunology*.

[34] Hunziker E. B. (2002). Articular Cartilage Repair: Basic Science and Clinical Progress. A Review of the Current Status and Prospects. *Osteoarthritis and Cartilage*.

[35] Tan Q., Lui P. P. Y., Rui Y. F., Wong Y. M. (2012). Comparison of Potentials of Stem Cells Isolated From Tendon and Bone Marrow for Musculoskeletal Tissue Engineering. *Tissue Engineering Part A*.

[36] Toh W. S., Lee E. H., Cao T. (2011). Potential of Human Embryonic Stem Cells in Cartilage Tissue Engineering and Regenerative Medicine. *Stem Cell Reviews and Reports*.

[37] Wong K. L., Lee K. B. L., Tai B. C., Law P., Lee E. H., Hui J. H. P. (2013). Injectable Cultured Bone Marrow-Derived Mesenchymal Stem Cells in Varus Knees With Cartilage Defects Undergoing High Tibial Osteotomy: A Prospective, Randomized Controlled Clinical Trial With 2 Years’ Follow-Up. *Arthroscopy: The Journal of Arthroscopic & Related Surgery*.

[38] Zhang S., Jiang Y. Z., Zhang W. (2013). Neonatal Desensitization Supports Long-Term Survival and Functional Integration of Human Embryonic Stem Cell-Derived Mesenchymal Stem Cells in Rat Joint Cartilage Without Immunosuppression. *Stem Cells and Development*.

[39] Zavatti M., Beretti F., Casciaro F., Bertucci E., Maraldi T. (2020). Comparison of the Therapeutic Effect of Amniotic Fluid Stem Cells and Their Exosomes on Monoiodoacetate-Induced Animal Model of Osteoarthritis. *BioFactors*.

[40] Wong K. L., Zhang S., Wang M. (2020). Intra-Articular Injections of Mesenchymal Stem Cell Exosomes and Hyaluronic Acid Improve Structural and Mechanical Properties of Repaired Cartilage in a Rabbit Model. *Arthroscopy: The Journal of Arthroscopic & Related Surgery*.

[41] Barile L., Vassalli G. (2017). Exosomes: Therapy Delivery Tools and Biomarkers of Diseases. *Pharmacology & Therapeutics*.

[42] Kourembanas S. (2015). Exosomes: Vehicles of Intercellular Signaling, Biomarkers, and Vectors of Cell Therapy. *Annual Review of Physiology*.

[43] Cai H. Q., Miao M. Y., Zhang W. L. (2020). AT1/2R Affects the Proliferation and Apoptosis of Chondrocytes Induced by Oxygen-Glucose Deprivation. *Bratislava Medical Journal*.

[44] Wei J., You G., Cheng H., Gao C. (2023). SPRED2 Promotes Autophagy and Attenuates Inflammatory Response in IL-1*β* Induced Osteoarthritis Chondrocytes via Regulating the p38 MAPK Signaling Pathway. *Tissue and Cell*.

[45] Shakibaei M., Mobasheri A., Buhrmann C. (2011). Curcumin Synergizes With Resveratrol to Stimulate the MAPK Signaling Pathway in Human Articular Chondrocytes in Vitro. *Genes & Nutrition*.

[46] Xia P., Ren S., Lin Q. (2015). Low-Intensity Pulsed Ultrasound Affects Chondrocyte Extracellular Matrix Production via an Integrin-Mediated p38 MAPK Signaling Pathway. *Ultrasound in Medicine & Biology*.

[47] Zhao J., Sun Y., Sheng X. (2023). Hypoxia-Treated Adipose Mesenchymal Stem Cell-Derived Exosomes Attenuate Lumbar Facet Joint Osteoarthritis. *Molecular Medicine*.

[48] Hammad M., Veyssiere A., Leclercq S., Patron V., Baugé C., Boumédiene K. (2023). Hypoxia Differentially Affects Chondrogenic Differentiation of Progenitor Cells From Different Origins. *International Journal of Stem Cells*.

[49] Peng Y., Jiang H., Zuo H.-D. (2023). Factors Affecting Osteogenesis and Chondrogenic Differentiation of Mesenchymal Stem Cells in Osteoarthritis. *World Journal of Stem Cells*.

[50] Li H., Li X., Jing X. (2018). Hypoxia Promotes Maintenance of the Chondrogenic Phenotype in Rat Growth Plate Chondrocytes Through the HIF-1*α*/YAP Signaling Pathway. *International Journal of Molecular Medicine*.

[51] Bin-Bin Z., Da-Wa Z. X., Chao L. (2022). M2 Macrophagy-Derived Exosomal miRNA-26a-5p Induces Osteogenic Differentiation of Bone Mesenchymal Stem Cells. *Journal of Orthopaedic Surgery and Research*.

[52] Silva A. M., Almeida M. I., Teixeira J. H. (2017). Dendritic Cell-Derived Extracellular Vesicles Mediate Mesenchymal Stem/Stromal Cell Recruitment. *Scientific Reports*.

[53] Ageta H., Ageta-Ishihara N., Hitachi K. (2018). UBL3 Modification Influences Protein Sorting to Small Extracellular Vesicles. *Nature Communications*.

[54] Singh V., Singh L. C., Vasudevan M. (2015). Esophageal Cancer Epigenomics and Integrome Analysis of Genome-Wide Methylation and Expression in High Risk Northeast Indian Population. *OMICS: A Journal of Integrative Biology*.

[55] Zhao X., Yongchun Z., Qian H. (2020). Identification of a Potential Tumor Suppressor Gene, UBL3, in Non-Small Cell Lung Cancer. *Cancer Biology and Medicine*.

[56] Liu H., Wilson K. R., Firth A. M. (2022). Ubiquitin-Like Protein 3 (UBL3) Is Required for MARCH Ubiquitination of Major Histocompatibility Complex Class II and CD86. *Nature Communications*.

[57] Houard X., Goldring M. B., Berenbaum F. (2013). Homeostatic Mechanisms in Articular Cartilage and Role of Inflammation in Osteoarthritis. *Current Rheumatology Reports*.

[58] Fioravanti A., Tenti S., McAllister M. (2019). Exploring the Involvement of NLRP3 and IL-1*β* in Osteoarthritis of the Hand: Results From a Pilot Study. *Mediators of Inflammation*.

[59] Jin C., Frayssinet P., Pelker R. (2011). NLRP3 Inflammasome Plays a Critical Role in the Pathogenesis of Hydroxyapatite-Associated Arthropathy. *Proceedings of the National Academy of Sciences of the United States of America*.

[60] Place D. E., Kanneganti T.-D. (2018). Recent Advances in Inflammasome Biology. *Current Opinion in Immunology*.

[61] Cai D., Yin S., Yang J., Jiang Q., Cao W. (2015). Histone Deacetylase Inhibition Activates Nrf2 and Protects Against Osteoarthritis. *Arthritis Research & Therapy*.

[62] Vaamonde-García C., Loureiro J., Valcárcel-Ares M. N. (2017). The Mitochondrial Inhibitor Oligomycin Induces an Inflammatory Response in the Rat Knee Joint. *BMC Musculoskeletal Disorders*.

[63] Chen Z., Zhong H., Wei J. (2019). Inhibition of Nrf2/HO-1 Signaling Leads to Increased Activation of the NLRP3 Inflammasome in Osteoarthritis. *Arthritis Research & Therapy*.

