# Supplementary material for: Bone Mesenchymal Stromal Cell-Derived Extracellular Vesicles Protect Articular Cartilage Through Regulating tRF-Gln-TTG-019/UBL3
Source: Mediators Inflamm. 2025 Jun 13;2025:2705953. doi: 10.1155/mi/2705953 (PMC12181665; doi:10.1155/mi/2705953)
Supplement: Supporting Information 1 — Table S1. Primer sequences. [file 2705953.f1.docx]

**Table S1. Primer sequences**

| ID | Sequence(5’- 3’) |
| --- | --- |
| U6 F | CTCGCTTCGGCAGCACA |
| U6 R | AACGCTTCACGAATTTGCGT |
| 18S.F | CGGTTCTATTTTGTTGGTTTTCG |
| 18S.R | TGCTTTCGCTCTGGTTCGTCTTG |
| tRF-Gln-TTG-019 | GGTCCCATGGTGTAATGGTTAGCACTCTGG |
| tRF-Gln-TTG-019 RT | CTCAACTGGTGTCGTGGAGTCGGCAATTCAGTTGAGCCAGAGT |
| tRF-Gln-TTG-019 F | ACACTCCAGCTGGGGGTCCCATGGTGTAATGGTTAGCACT |
| GAPDH.F | TGTTCGTCATGGGTGTGAAC |
| GAPDH.R | ATGGCATGGACTGTGGTCAT |
| UBL3.F | AGTAATGTCCCGGCGGATATG |
| UBL3.R | TCAGAAGCAGAATCGTTAGGAGA |
| tRF-Gln-TTG-025 | GGTCTCATGGTGTAATGGTTAGCACTCTGG |
| tRF-Gln-TTG-025 RT | CTCAACTGGTGTCGTGGAGTCGGCAATTCAGTTGAGCCAGAGT |
| tRF-Gln-TTG-025 F | ACACTCCAGCTGGGGGTCTCATGGTGTAATGGTTAGCACT |
| tRF-Gln-TTG-019 | GGTCCCATGGTGTAATGGTTAGCACTCTGG |
| tRF-Gln-TTG-019 RT | CTCAACTGGTGTCGTGGAGTCGGCAATTCAGTTGAGCCAGAGT |
| tRF-Gln-TTG-019 F | ACACTCCAGCTGGGGGTCCCATGGTGTAATGGTTAGCACT |
| tRF-Pro-TGG-009 | GGCTCGTTGGTCTAGGGGTATGATTCTCGG |
| tRF-Pro-TGG-009 RT | CTCAACTGGTGTCGTGGAGTCGGCAATTCAGTTGAGCCGAGAA |
| tRF-Pro-TGG-009 F | ACACTCCAGCTGGGGGCTCGTTGGTCTAGGGGTATGATTC |
| tRF-Pro-AGG-021 | GGCTCGTTGGTCTAGGGGTATGATTCTCGC |
| tRF-Pro-AGG-021 RT | CTCAACTGGTGTCGTGGAGTCGGCAATTCAGTTGAGGCGAGAA |
| tRF-Pro-AGG-021 F | ACACTCCAGCTGGGGGCTCGTTGGTCTAGGGGTATGATTC |
| tRF-Tyr-GTA-026 | TCCGGCTCGAAGGACCA |
| tRF-Tyr-GTA-026 RT | CTCAACTGGTGTCGTGGAGTCGGCAATTCAGTTGAGTGGTCCT |
| tRF-Tyr-GTA-026 F | ACACTCCAGCTGGGTCCGGCTCGAAGG |
| tRF-Arg-TCT-011 | GGCTCCGTGGCGCAATGGATAGCGCATTG |
| tRF-Arg-TCT-011 RT | CTCAACTGGTGTCGTGGAGTCGGCAATTCAGTTGAGCAATGCG |
| tRF-Arg-TCT-011 F | ACACTCCAGCTGGGGGCTCCGTGGCGCAATGGATAGCGC |
| tRF-Arg-TCT-014 | GGCTCTGTGGCGCAATGGATAGCGCATTG |
| tRF-Arg-TCT-014 RT | CTCAACTGGTGTCGTGGAGTCGGCAATTCAGTTGAGCAATGCG |
| tRF-Arg-TCT-014 F | ACACTCCAGCTGGGGGCTCTGTGGCGCAATGGATAGCGC |
| tRF-Glu-CTC-017 | TCCCTGGTGGTCTAGTGGTTAGGATTCAG |
| tRF-Glu-CTC-017 RT | CTCAACTGGTGTCGTGGAGTCGGCAATTCAGTTGAGCTGAATC |
| tRF-Glu-CTC-017 F | ACACTCCAGCTGGGTCCCTGGTGGTCTAGTGGTTAGGAT |
| tRF-Val-AAC-025 | GTTTCCGTAGTGTAGTGGTTATCACATTC |
| tRF-Val-AAC-025 RT | CTCAACTGGTGTCGTGGAGTCGGCAATTCAGTTGAGGAATGTG |
| tRF-Val-AAC-025 F | ACACTCCAGCTGGGGTTTCCGTAGTGTAGTGGTTATCAC |
| tRF-His-GTG-026 | GCCGTGATCGTATAGTGGTTAGTACTCTG |
| tRF-His-GTG-026 RT | CTCAACTGGTGTCGTGGAGTCGGCAATTCAGTTGAGCAGAGTA |
| tRF-His-GTG-026 F | ACACTCCAGCTGGGGCCGTGATCGTATAGTGGTTAGTAC |
