# Supplementary material for: Bone Mesenchymal Stromal Cell-Derived Extracellular Vesicles Protect Articular Cartilage Through Regulating tRF-Gln-TTG-019/UBL3
Source: Mediators Inflamm. 2025 Jun 13;2025:2705953. doi: 10.1155/mi/2705953 (PMC12181665; doi:10.1155/mi/2705953)
Supplement: Supporting Information 2 — Table S2. Sequences of inhibitor, mimics and shRNA. [file 2705953.f2.docx]

**Table S2. Sequences of inhibitor, mimics and shRNA.**

| ID | Sequence(5’- 3’) |
| --- | --- |
| tRF-Gln-TTG-019 inhibitor sense | GGTCTCACGATTGGTAATGTGGTACCCTGG |
| tRF-Gln-TTG-019 mimics sense | GGTCCCATGGTGTAATGGTTAGCACTCTGG |
| miR- inhibitor negative control (NC) sense | CGGCTATTCGGGCATTTGCC |
| miR- mimics negative control (NC) sense | GGCTCATGCCCGAATAGCCG |
| negative control shRNA (shNC) sense | AGTTATAATGAGTTTTATTG |
| negative control shRNA (shNC)  antisense | CAATAAAACTCATTATAACT |
| UBL3 shRNA sense | GCCAATGGACTGGGAAGAAGA |
| UBL3 shRNA antisense | TCTTCTTCCCAGTCCATTGGC |
