# Supplementary figures and images for: Bone Mesenchymal Stromal Cell-Derived Extracellular Vesicles Protect Articular Cartilage Through Regulating tRF-Gln-TTG-019/UBL3
Source: Mediators Inflamm. 2025 Jun 13;2025:2705953. doi: 10.1155/mi/2705953 (PMC12181665; doi:10.1155/mi/2705953)

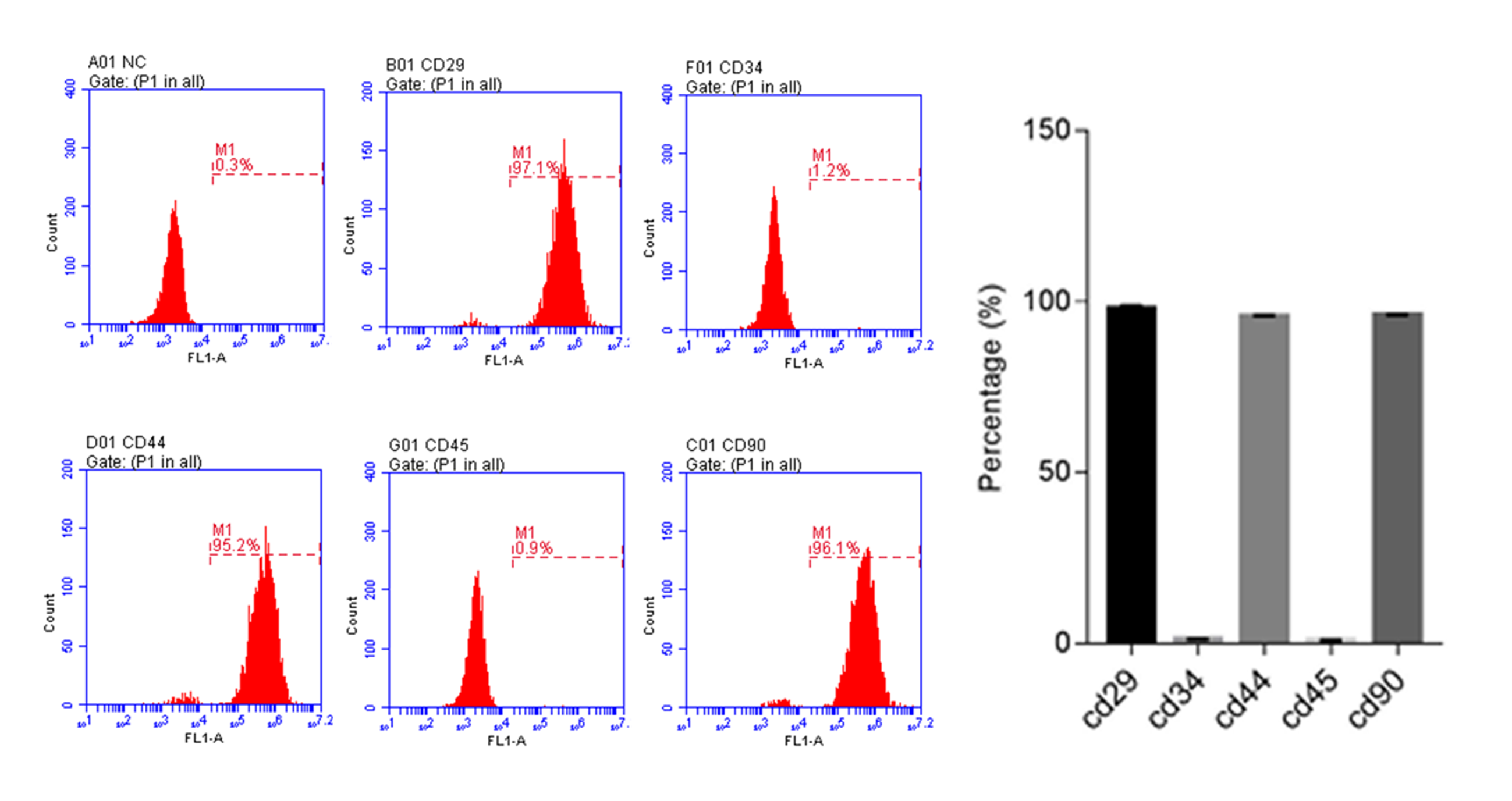

Supplement: Supporting Information 4 — Figure S1. Identification of rat BMSCs. Rat BMSCs were isolated and performed flow cytometry assay (FITC labeled CD29, CD34, CD44, CD45, and CD90). Negative control (NC) was incubated with PBS. [file 2705953.f4.tif]

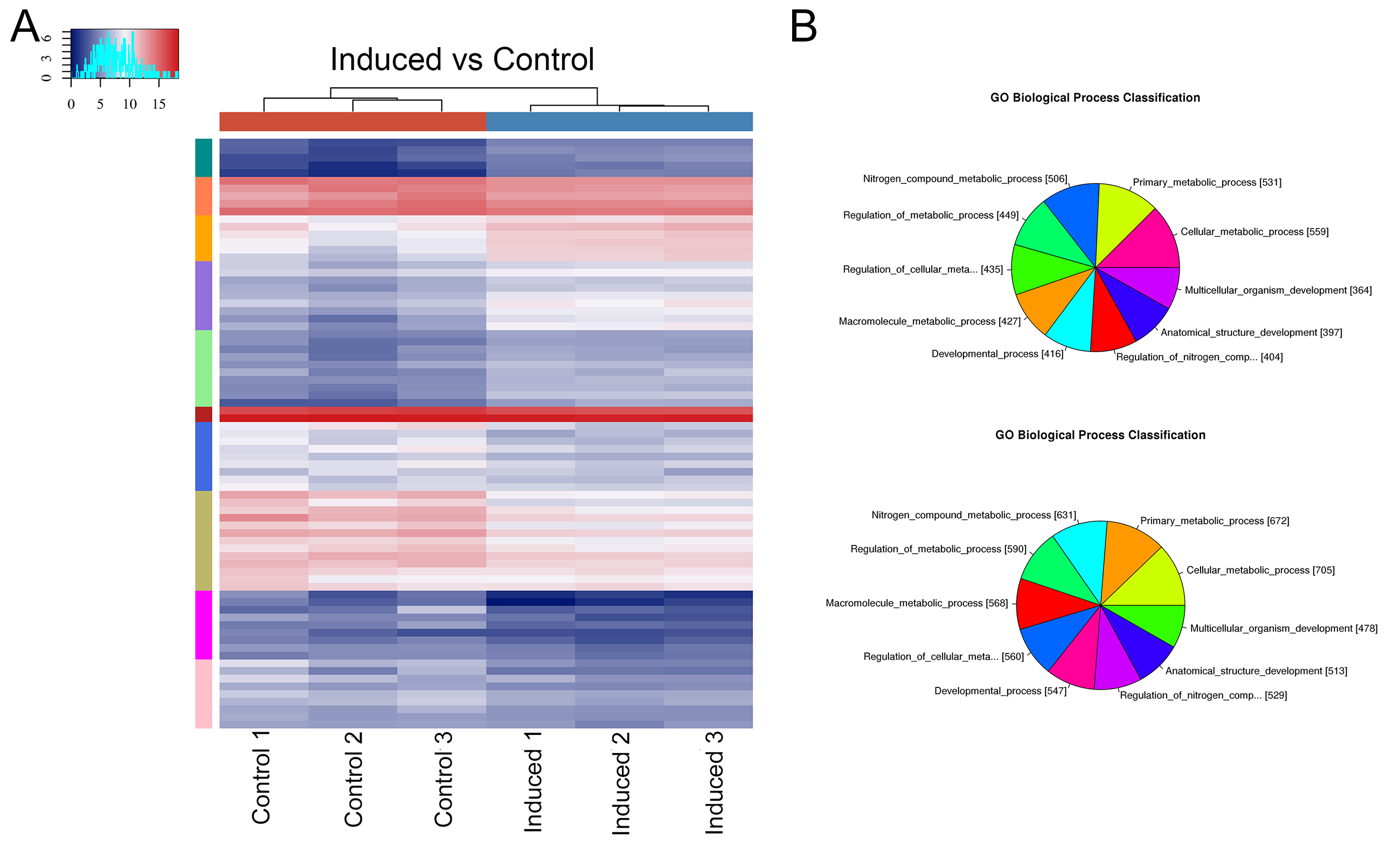

Supplement: Supporting Information 5 — Figure S2. Heat map and GO TERM assay. (A) Differentially expressed tRFs were displayed by heat map. The color in the panel represented the relative expression level (log2-transformed). The color scale is shown below: blue represented an expression level below the mean, and red represented an expression level above the mean. The colored bar top at the top panel showed the sample group, and the colored bar at the right side of the panel indicated the divisions which were performed using K-means. (B) GO TERM analysis in up (upper panel) or down-regulated (lower panel) tRFs. [file 2705953.f5.tif]

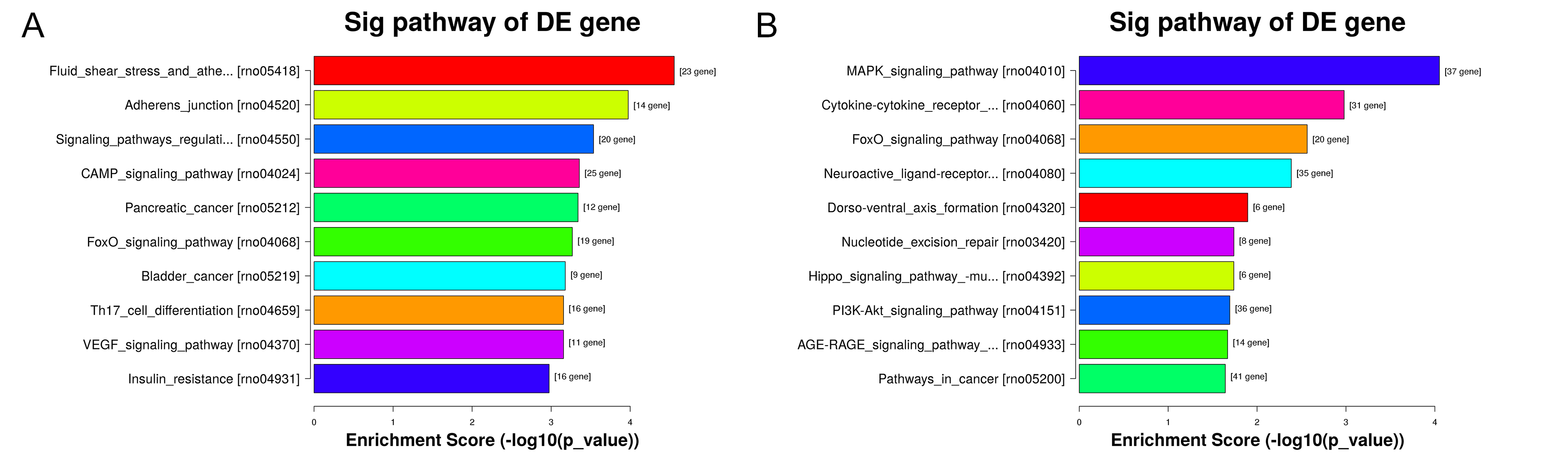

Supplement: Supporting Information 6 — Figure S3. Signaling pathway assay. (A and B) Signal pathway analysis of target genes in up (left panel) or down-regulated (right panel) tRFs. [file 2705953.f6.tif]

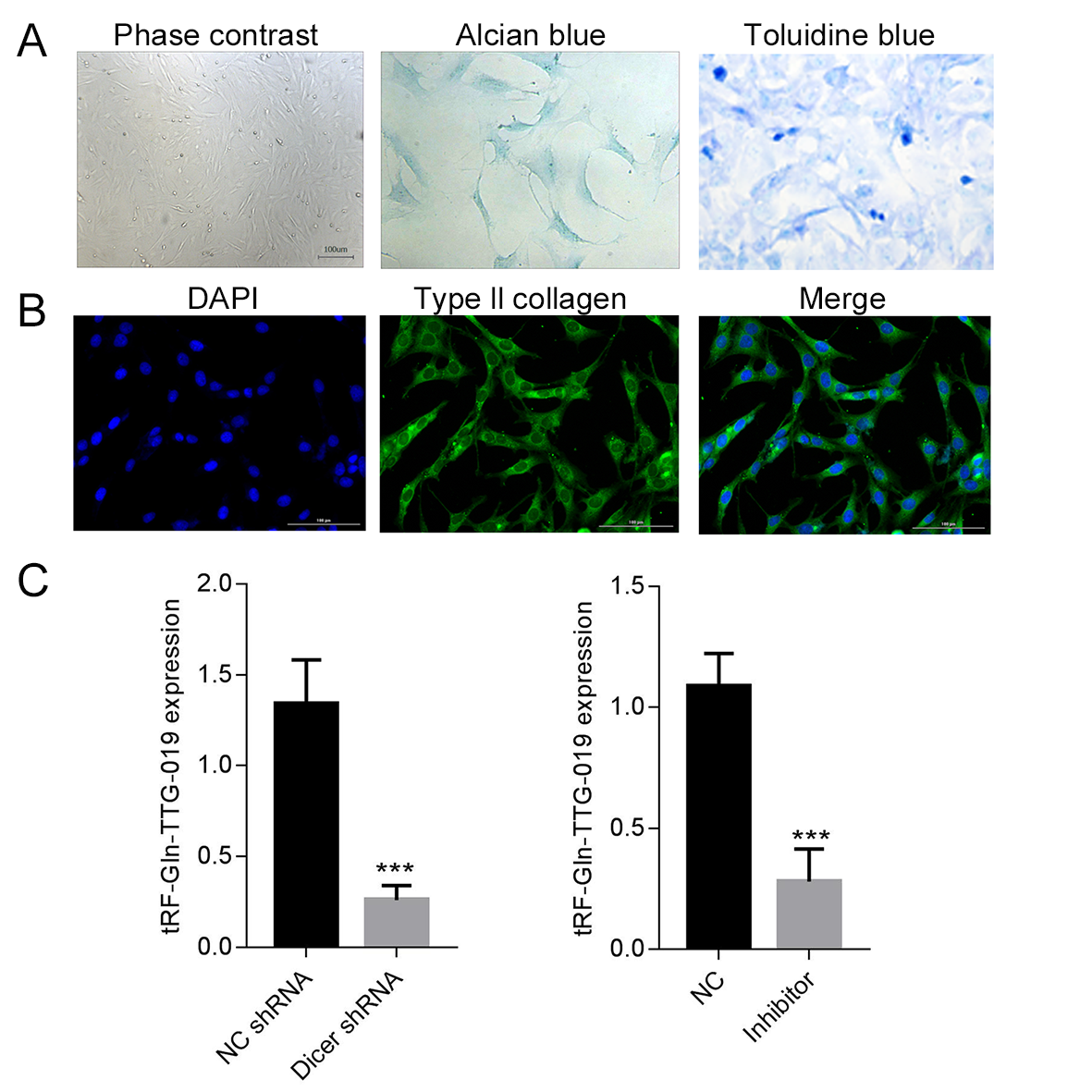

Supplement: Supporting Information 7 — Figure S4. Suppression of tRF-Gln-TTG-019 expression by the specific inhibitor. (A and B) Primary rat chondrocytes were isolated from knee cartilage and identified by Toluidine blue staining and type collagen II by immunofluorescence. (C) Chondrocytes were transfected with NC shRNA or Dicer shRNA. The expression of tRF-Gln-TTG-019 was detected by real-time PCR (left panel). Rat chondrocytes were transfected with NC or tRF-Gln-TTG-019 inhibitor, the expression of tRF-Gln-TTG-019 was detected by real-time PCR (right panel). ⁣∗∗∗p < 0.001. [file 2705953.f7.tif]

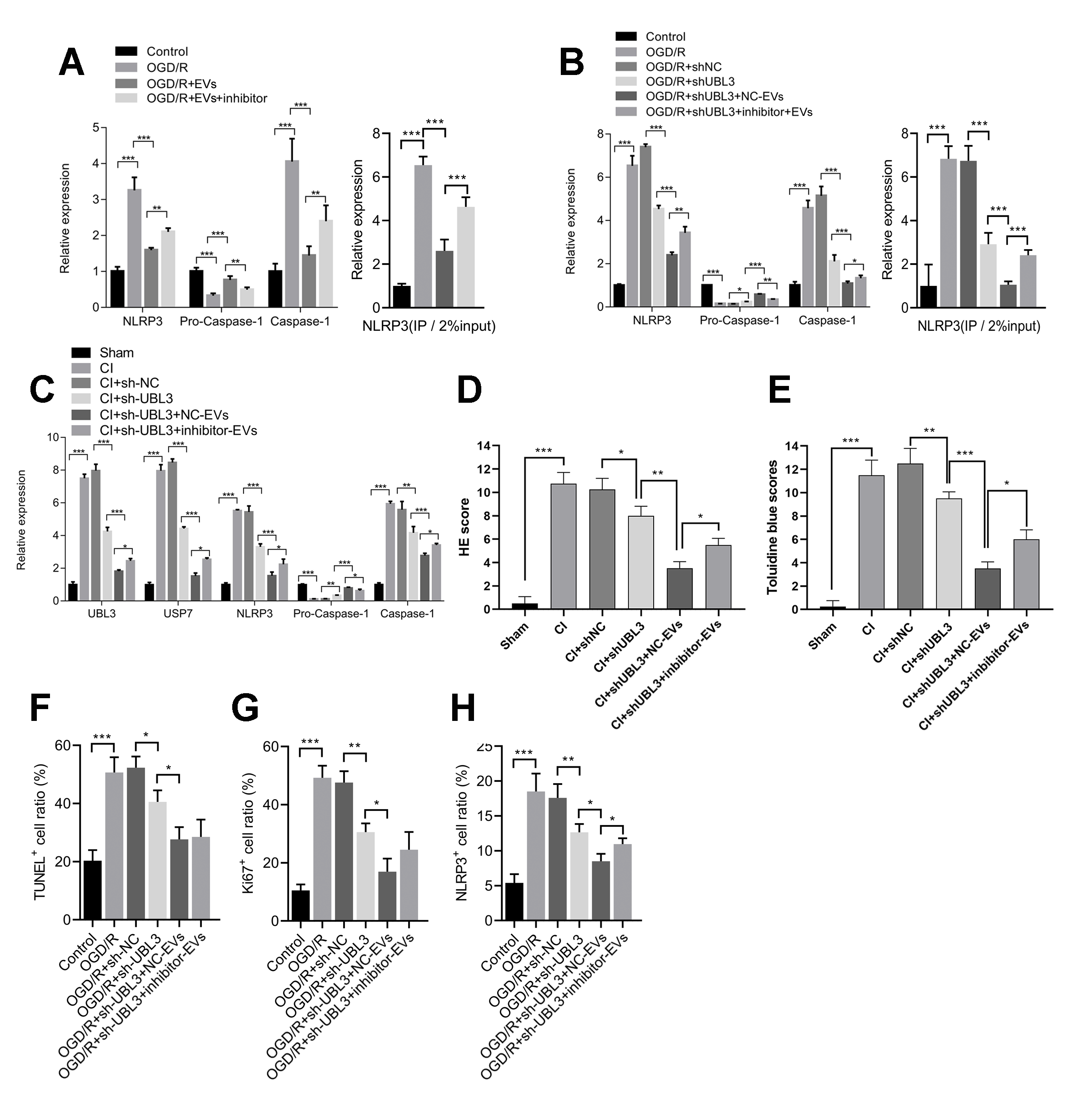

Supplement: Supporting Information 8 — Figure S5. Statistical analysis of Western blot and IHC. (A) The protein expressions were quantified from three independent experiments. The statistical results were shown. A was related to Figure 3J,K; B was related to Figure 5G,H; C was related to Figure 6G. (D and E) The scoring of H&E and Toluidine blue in Figure 6A,B. (F and H) Quantified data of TUNEL staining and IHC results in Figure 6C–E. ⁣∗p < 0.05; ⁣∗∗p < 0.01; ⁣∗∗∗p < 0.001. [file 2705953.f8.tif]
