# Supplementary material for: Bone Mesenchymal Stromal Cell-Derived Extracellular Vesicles Protect Articular Cartilage Through Regulating tRF-Gln-TTG-019/UBL3
Source: Mediators Inflamm. 2025 Jun 13;2025:2705953. doi: 10.1155/mi/2705953 (PMC12181665; doi:10.1155/mi/2705953)
Supplement: Supporting Information 9 — Detailed description of some experimental methods. [file 2705953.f9.docx]

**Supplementary method**

***Alcian blue staining***

The cells were fixed with 4% paraformalydehyde for 30 min, and then washed with phosphate-buffered saline (PBS). The dye alcian blue (0.1% stock solution) was added for 30 mins incubation at room temperature and washed with distilled water. The images were observed by optical microscope (Leica DMI 3000B; Leica Microsystems, Germany).

***RNA extraction***

According to the manufacturer's instructions, the total RNA was extracted from the cells or EVs by using TRIzol reagent according to the manufacturer's instructions (ThermoFisher Scientific). The quantity of RNA was measured using a NanoDrop 2000 spectrophotometer (ThermoFisher Scientific).

***Immunofluorescence assay***

The cells were seeded on 12 well plates and treated according to the following procedure: cells were fixed with 4% paraformaldehyde for 10 minutes and permeabilized with 0.1% TritonX-100. Then, block the cells with goat serum at room temperature for one hour. Finally, they were incubated with indicated antibodies overnight at 4°C. The next day, secondary antibodies and DAPI (Boster, Wuhan, China), which was used to characterize the nuclei of chondrocytes, were added to the cells and incubated them at room temperature for one hour. At last, the cells were observed under the fluorescent microscope (Leica).

***Cell Counting Kit 8 (CCK8) assay***

Cells were seeded into a 96-well plate and treated according to the following procedure: the culture media was removed, and 10 μL of CCK8 solution (5 mg/mL) (Beyotime) was added to the cells. After incubating the cells at 37°C for 2 hours, we added 200 μL of dimethyl sulfoxide (DMSO) solution to each well. for incubation for 15 min. At last, the optical density OD value of cells was determined at 490 nm by multimode plate reader.

***EdU staining***

Twelve hours before collecting the cells, they were treated with a 10 μM EdU stain (Solarbio, Beijing, China). The cells were fixed with 4% paraformaldehyde for 15 min, then treated with 5% glycine for 5 min. After washing the cells with PBS, 0.5% Triton X-100 was used to permeabilize the cells. Finally, we incubated the cells with 300 μL of 1×Apollo fluorescent dyeing liquid for 30 min. The images of cells were observed with a fluorescence microscope (Leica).

***Flow cytometry analysis***

Cell apoptosis was determined with an Annexin V-FITC/PI kit (Beyotime). The cells were treated according to the procedure described in our previous study. Then, wash the cells with PBS and stain with 10 μL of Annexin V-FITC and 5 μL of PI for 15 min at room temperature. The staining procedure was performed completely in the dark. The apoptosis rate was analyzed with a FACS Calibur flow cytometer (BD, New York, NJ, USA).

***Tunel assay***

The cells were fixed with formaldehyde for 15 min in an ice bath. An ice-cold 70% ethanol was added to the cells for incubation for 30 mins after washing the cells with PBS. Then, the cells were spun down and washed with a washing buffer. Afterwards, resuspend the cells in a staining solution and incubate for 60 min at 37°C. The cells were incubated with PI/RNAse A solution for another 30 min. At last, the images were observed with the help of a fluorescence microscope.

***ELISA test***

The supernatant of the cell culture or rat synovial fluid was collected and analyzed by the ELISA test. The samples were treated according to a procedure described in the commercial kits (MEIMIAN, Shanghai, China; MM-0047R1, MM-0194R1, MM-0873R2). Briefly, the samples were diluted and added carefully to plates, which were then incubated at 37 ºC for one hour. After washing the plates five times with washing buffer, we added chromogenic agents to the plates and incubated them for 30 min at 37ºC. The results were calculated after measuring the value of optical density (OD) at a fixed wavelength of 450 nm with a microplate reader (ThermoFisher Scientific).
